# Mechanical ventilation in patients with acute brain injury: a systematic review with meta-analysis

**DOI:** 10.1186/s13054-023-04509-3

**Published:** 2023-06-06

**Authors:** Karim Asehnoune, Paul Rooze, Chiara Robba, Marwan Bouras, Luciana Mascia, Raphaël Cinotti, Paolo Pelosi, Antoine Roquilly

**Affiliations:** 1 Nantes Université, CHU Nantes, Pôle Anesthésie Réanimations, Service d’Anesthésie Réanimation Chirurgicale, Hôtel Dieu, 44093 Nantes, France; 2 Anesthesia and Critical Care, San Martino Policlinico Hospital, University of Genoa, Genoa, Italy; 3 Dipartimento di Scienze Biomediche e Neuromotorie, University of Bologna, Bologna, Italy; 4 Nantes Université, Univ Tours, CHU Nantes, CHU Tours, INSERM, MethodS in Patients-Centered Outcomes and HEalth Research, SPHERE, 44000 Nantes, France; 5 Nantes Université, INSERM, Center for Research in Transplantation and Translational Immunology, UMR 1064, 44000 Nantes, France; 6 Department of Anaesthesia and Critical Care, Hôtel-Dieu, University Hospital of Nantes, 1 Place Alexis Ricordeau, 44093 Nantes, France

## Abstract

**Objective:**

To describe the potential effects of ventilatory strategies on the outcome of acute brain-injured patients undergoing invasive mechanical ventilation.

**Design:**

Systematic review with an individual data meta-analysis.

**Setting:**

Observational and interventional (before/after) studies published up to August 22nd, 2022, were considered for inclusion. We investigated the effects of low tidal volume Vt < 8 ml/Kg of IBW versus Vt >  = 8 ml/Kg of IBW, positive end-expiratory pressure (PEEP) < or >  = 5 cmH_2_O and protective ventilation (association of both) on relevant clinical outcomes.

**Population:**

Patients with acute brain injury (trauma or haemorrhagic stroke) with invasive mechanical ventilation for ≥ 24 h.

**Main outcome measures:**

The primary outcome was mortality at 28 days or in-hospital mortality. Secondary outcomes were the incidence of acute respiratory distress syndrome (ARDS), the duration of mechanical ventilation and the partial pressure of oxygen (PaO_2_)/fraction of inspired oxygen (FiO_2_) ratio.

**Results:**

The meta-analysis included eight studies with a total of 5639 patients. There was no difference in mortality between low and high tidal volume [Odds Ratio, OR 0.88 (95%Confidence Interval, CI 0.74 to 1.05), *p* = 0.16, *I*^2^ = 20%], low and moderate to high PEEP [OR 0.8 (95% CI 0.59 to 1.07), *p* = 0.13, *I*^2^ = 80%] or protective and non-protective ventilation [OR 1.03 (95% CI 0.93 to 1.15), *p* = 0.6, *I*^2^ = 11]. Low tidal volume [OR 0.74 (95% CI 0.45 to 1.21, *p* = 0.23, *I*^2^ = 88%], moderate PEEP [OR 0.98 (95% CI 0.76 to 1.26), *p* = 0.9, *I*^2^ = 21%] or protective ventilation [OR 1.22 (95% CI 0.94 to 1.58), *p* = 0.13, *I*^2^ = 22%] did not affect the incidence of acute respiratory distress syndrome. Protective ventilation improved the PaO_2_/FiO_2_ ratio in the first five days of mechanical ventilation (*p* < 0.01).

**Conclusions:**

Low tidal volume, moderate to high PEEP, or protective ventilation were not associated with mortality and lower incidence of ARDS in patients with acute brain injury undergoing invasive mechanical ventilation. However, protective ventilation improved oxygenation and could be safely considered in this setting. The exact role of ventilatory management on the outcome of patients with a severe brain injury needs to be more accurately delineated.

**Supplementary Information:**

The online version contains supplementary material available at 10.1186/s13054-023-04509-3.

## Introduction

Acute brain injury (BI) is estimated to affect 100 million patients annually [1, 2], with high mortality rates prolonged and severe disability worldwide. In the most severe form, patients with BI require invasive mechanical ventilation. It is generally accepted that the evolution of neurological pathology mainly drives outcomes after BI; however, the influence of extracerebral organ dysfunction seems essential and remains a matter of debate. Patients with BI frequently develop respiratory complications, such as ventilator-associated pneumonia and acute distress respiratory syndrome, associated with increased ventilator time and poor outcomes [3]. In the general population of critically ill patients, accelerating the weaning from mechanical ventilation and implementing specific interventions to prevent lung injury is fundamental [4]; however, the optimal mechanical ventilation settings are still unclear in the population of BI patients. In the general intensive care unit (ICU) population, the use of high tidal volumes (Vt) and inspiratory pressures have been shown to overstretch the alveoli and to be the leading cause of ventilator-induced lung injury (VILI) [5, 6]. Currently, mechanical ventilation (MV) with low Vt and moderate to high positive end-expiratory pressure (PEEP), defined as a protective strategy, is recommended in patients with acute respiratory distress syndrome (ARDS) but also in patients with healthy lungs [7]. This may be important in patients with acute BI who generally have a longer duration of mechanical ventilation due to prolonged cognitive impairment, higher rates of hospital-acquired pneumonia and mortality compared to non-neurologic patients [8, 9]. However, the general application of the protective ventilation strategies is challenging in BI patients; although recent data suggest that the use of low Vt could improve the outcomes without causing any harm even in this population [10], protective ventilation strategy in patients with BI can increase carbon dioxide values and be detrimental on intracranial pressure and cerebral hemodynamic. Therefore, lung protective strategies have been poorly applied in ABI patients, and these patients have been generally excluded from the significant trials exploring the effect of these strategies on outcomes. The application of low PEEP and high or moderate Vt (to maintain normocapnia or moderate hypocapnia) is still common in this population [11]. Despite the lack of robust evidence, recent guidelines and expert recommendations suggest that lung protective strategies should be considered even in BI patients. We, therefore, conducted an individual data meta-analysis to assess the effect of protective ventilation strategies (i.e., low Vt and moderate to higher PEEP) on patient outcomes- i.e. decreased mortality and respiratory complication rates.

## Methods

### Systematic review

We adhered to the *Preferred Reporting Items for Systematic Reviews and Meta-Analysis-Protocols* (PRISMA-P) guidelines (Additional file 1: Table S1). The protocol of this study was not registered with the *International Prospective Register of Systematic Reviews* (PROSPERO).

### Data sources and search strategy

A systematic literature search was performed using the following databases to identify relevant studies: PubMed® (MEDLINE/Index Medicus), EMBASE (via Ovid), and the Cochrane Controlled Clinical trials register.

The Major Medical Subject Heading terms used for the search were “brain injury” and “Mechanical Ventilation”, with the limit “human” and “adult 18^+^ years”. The complete systematic review search string and strategy are reported in the Additional file (Additional file 1: Fig. S1). We included articles published up to August 22nd 2022, in scientific journals. Only articles in English and French were considered. Editorials, commentaries, letters to editor, opinion articles, reviews, and meeting abstracts, were also excluded, as well as original articles lacking abstract and/or quantitative details.

We attempted to select all relevant studies investigating the association between tidal volume, positive end-expiratory pressure, lung protective strategies and outcomes in brain-injured patients. The following outcomes were determined: mortality at 28-day or in-hospital mortality (as reported by authors), rate of acute respiratory distress syndrome, duration of mechanical ventilation and the partial pressure of oxygen (PaO_2_)/fraction of inspired oxygen (FiO_2_) ratio during the first 5 days. The references of all included papers, review articles, commentaries and editorials on this topic were also reviewed to identify other studies of interest that were missed during the primary search. When multiple publications of the same research group/centre described case series potentially overlapping were found, we used the more recent publication, if eligible.

Researchers accessing the primary data (KA and AR) reviewed individual study variables and extracted relevant standard variables into a single dataset. Hospital and 90-day mortality data were recoded into a single variable (“hospital mortality censored at 28 days”). There were no duplicate participants in the included studies.

### Definitions and outcomes

Low Vt was defined as Vt < 8 ml/Kg of Ideal Body Weight (IBW); low PEEP was defined as PEEP < 5 cm H_2_O ref for both; protective ventilation ref was defined as Vt < 8 ml/Kg of IBW and PEEP >  = 5 cm H_2_O. Different mechanical ventilation settings were compared: (1) Vt < 8 ml/Kg of IBW vs. Vt >  = 8 ml/Kg of IBW; (2) PEEP < or >  = 5 cm H_2_O; (3) Vt < 8 ml/Kg of IBW and PEEP >  = 5 cm H_2_O vs. Vt >  = 8 ml/Kg of IBW and PEEP < 5 cm H_2_O. The primary outcome was in-hospital or 28-day mortality. The secondary outcomes were PaO_2_/FiO_2_ during the first 5 days, mechanical ventilation duration, and ARDS risk.

The ARDS was defined according to the Berlin definition [6]. However, before 2012, the ARDS definition was left to the clinician’s discretion.

### Data extraction and quality assessment

Three examiners independently evaluated titles and abstracts. The articles were then subdivided into three subgroups: “included” and “excluded” (if the two examiners agreed with the selection) or “uncertain” (in case of disagreement). In the case of “uncertain” classification, discrepancies were resolved by further examination by three expert authors (KA, RC and AR), and no disagreement was observed.

We used a standardized electronic spreadsheet (Microsoft Excel, V 14.4.1; Microsoft, Redmond, WA) to extract the data from all included studies: study characteristics (*i.e.* number of sites, country), patient population (*i.e.* demographics, type of brain injury, baseline illness severity scores), monitoring and interventions (*i.e.* mechanical ventilation characteristics) and clinical outcomes. When necessary, the corresponding authors of the included studies were contacted to obtain missing data related to trial demographics, methods and outcomes.

### Assessment of risk of bias in the included studies

The internal validity of the included studies was assessed by two expert authors (KA and RC and discrepancies were resolved by a third author (AR) using the RoB 2: a revised Cochrane Collaboration’s risk-of-bias tool for randomized trials. The Rob 2 considers five bias domains: (1) the randomization process; (2) the deviations from intended interventions; (3) missing outcome data; (4) the measurement of the outcome; (5) the selection of the reported results. Finally, an overall risk of bias was calculated, and studies were included in either high-risk/ some concerns /low-risk groups. **(**Additional file 1: Table S2).

### Statistical analysis

Statistical analysis was conducted on the summary statistics described in the selected articles (e.g., means, medians, proportions), and, therefore, the statistical unit of observation for all the selected variables was the single study and not the patient. Descriptive statistics of individual studies used different statistical indicators for central tendency and variability, such as means and standard deviations, whereas absolute and relative frequencies were adopted for qualitative variables. To show one single indicator for the quantitative variables, we collected means with standard deviations (SD) or medians and inter-quartile ranges (IQR) were used, as appropriate.

Treatment effects were reported as relative risks, RRs with 95% confidence intervals for discontinuous outcomes, and weighted mean differences (WMD) with 95% CI for continuous data. We assessed publication bias using a funnel plot for the considered outcomes. Statistical heterogeneity and inconsistency were measured using Q and *I*^2^ tests and were considered significant when *p* < 0.1 and *I*^2^ > 50%. According to heterogeneity, random or fixed effect models were used to perform metanalysis. According to Borenstein et al. [12], *I*^2^ values around 25, 50, and 75% were considered to represent respectively low, moderate, and severe statistical inconsistency. Analyses were performed using RevMan® version 5.3 using random-effects models with fixed-effects models for comparison.

The time course of PaO2/FiO2 was analyzed by two-way ANOVA, considering death a competitive event.

## Results

### Review of literature and meta-analysis

The electronic search identified 96 titles after removing duplicate studies. The three experts (KA, RC and AR) independently assessed articles for study inclusion using the Preferred Reporting Items for Systematic review and Meta-Analysis Protocols (PRISMA-P) for data reporting. The systematic review of the literature identified seven studies and one cohort, “Atlanrea" [13–15], including the current results, that involved a total of 5639 patients providing in-hospital and day-28 mortality. Among the eight studies included, 3 are retrospective observational multicenter studies [9, 32, 33], 2 are interventional before-and-after studies [3, 10], one randomized controlled trial [36], one posthoc analysis of a prospective observational study [24], and one unpublished database (Cohort Atlanréa) (Additional file 1: Tables S2 and S3). The demographic characteristics of the study population are presented in Table 1. Nevertheless, there is a lack of demographic data from the Atlanrea cohort and Pelosi study [9]

**Table 1:** Demographic characteristics form the 7 published studies

	Population characteristics
	**N = 5639**
Age, years, *means (SD)*	54.9 (18.5)
Sex M/F ***(%)***	2424 / 1551 (61% male)
Pathology (N = 3980), ***N (%)***	
Traumatic brain injury	1482 (37.3)
Subarachnoid haemorrhage	552 (13.9)
Stroke	1451 (36.5)
Others or not reported	490 (12.3)
Glasgow Coma Scale, (N = 3792) *means (SD)*	7 (3)
***Intra-Cranial Hypertension, N (%)***	962/3816 (25.2)
***First ICP, (N* = *1212) means (SD)***	15.8 (14.3)
Decompressive craniectomy***, N (%)***	117/1243 (9.4)
Pa/FiO2 ratio at baseline (n = 3792)	355 (119)
Ventilator setting	
Tidal volume, ml/Kg of IBW, *mean (SD)*	8.6 (1.9)
Tidal volume < 8, ml/Kg of IBW, *N (%)*	1732 / 3669 (47.2)
***Groupe High Vt, mean (SD)***	9.6 (1.6)
***Groupe Low Vt, mean (SD)***	6.9 (0.7)
Tidal volume missing data, *N (%)*	304 (7.6)
PEEP, cmH_2_O, *mean (SD)*	4.7 (1.8)
PEEP ≥ 5 cmH_2_O, *N (%)*	1341 (50.9)
***Group High PEEP, mean (SD)***	5.4 (0.9)
***Group Low PEEP, mean (SD)***	2.1 (1.6)
PEEP missing data, *N (%)*	14 (0.1)
Acute respiratory distress syndrome, *N (%)*	881 / 5639 (15.6)
Duration of mechanical ventilation (days), *mean (SD)*	14.6 (11.6)
Death, *N (%)*	1675/ 5139 (32.6)

## Population

The mean age of the included patients was 54.9 (18.5) years, and 61% were males. Patients majorly suffered from trauma (37.3%) and stroke with 36.5%. The mean Glasgow coma scale was 7 (3), and the mean intracranial pressure at baseline was 15.8 (14.3) mmHg. Among the 3816 patients with available data, 962 (25.2%) developed one or more episodes of intracranial hypertension. Tidal volumes were lower than 8 ml/kg of IBW in 47% of patients, and PEEP was > 5 in 50.9%. The mean Vt was 9.6 (1.6) ml/kg of IBW in the high Vt Group and 6.9 (0.7) ml/kg of IBW in the Low Vt Group. The level of PEEP was 5.4 (0.9) mmH2O in the high PEEP group and 2.1 (1.6) mmH2O in the low PEEP group. (Table 1).

### Primary outcome

**Fig. 1:**
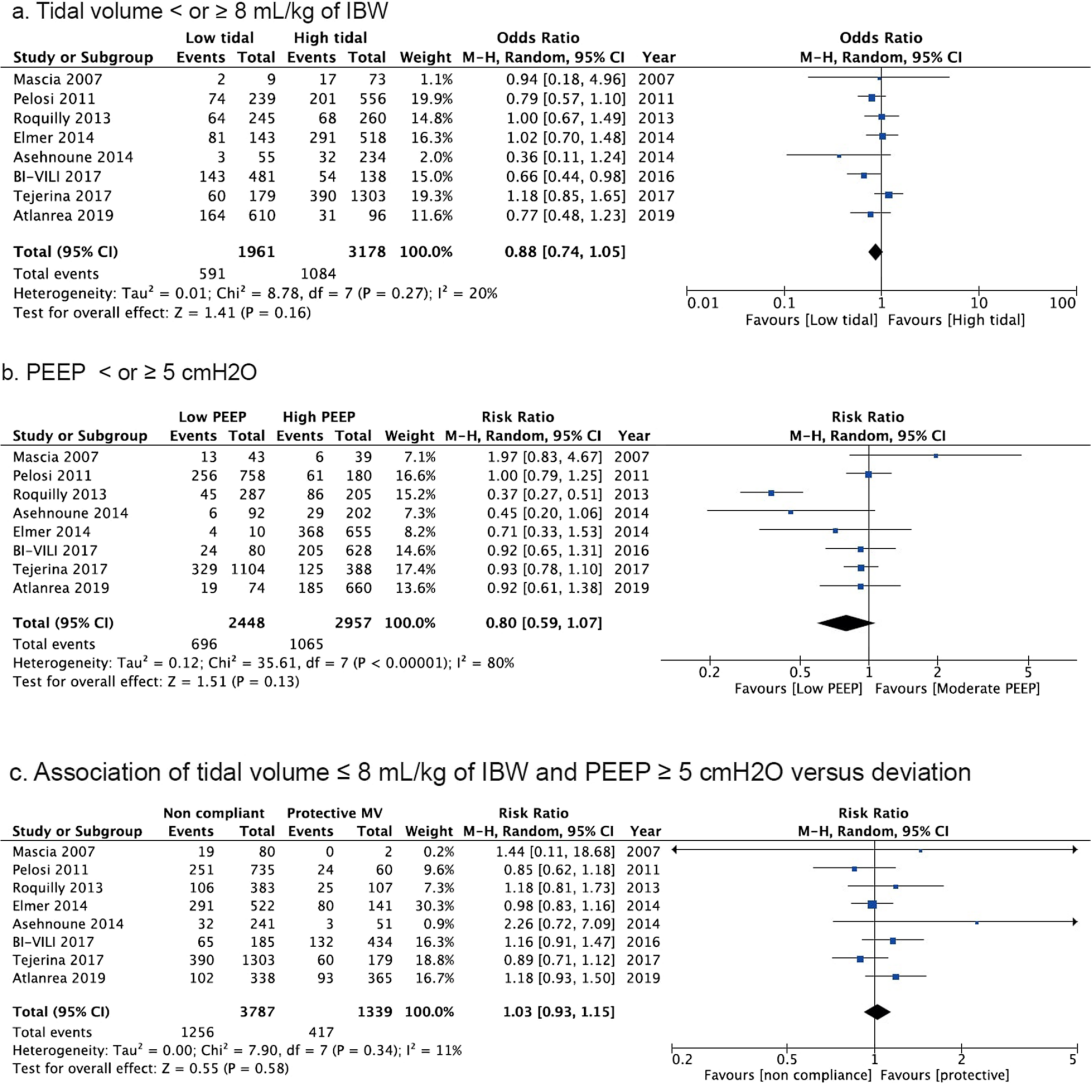
In-hospital mortality or 28-day mortality according to ventilator settings

Low Vt compared to high Vt (intervention, 591/1961 deaths [30.1%] vs. control, 1084/3178 deaths [34.1%]; OR 0.88 (95% CI 0.74 to 1.05), *p* = 0.16, *I*^2^ = 20%, Fig. 1a) as well as low PEEP compared to moderate to high PEEP (low PEEP 696/2448 deaths [28.4%] vs. moderate PEEP 1065/2957 deaths [36%]; OR 0.8 (95% CI 0.59 to 1.07, *p* = 0.13, *I*^2^ = 80%, Fig. 1b) were not associated with in-hospital mortality. A protective ventilation strategy associating low Vt and moderate to high PEEP was not associated with improved primary outcome (intervention 1256/3787 [33.1%] vs. control 417/1339 [31.1%], OR 1.03 (95% CI 0.93 to 1.15), *p* = 0.58, *I*^2^ = 11%, Fig. 1c).

### Secondary outcomes

**Fig. 2:**
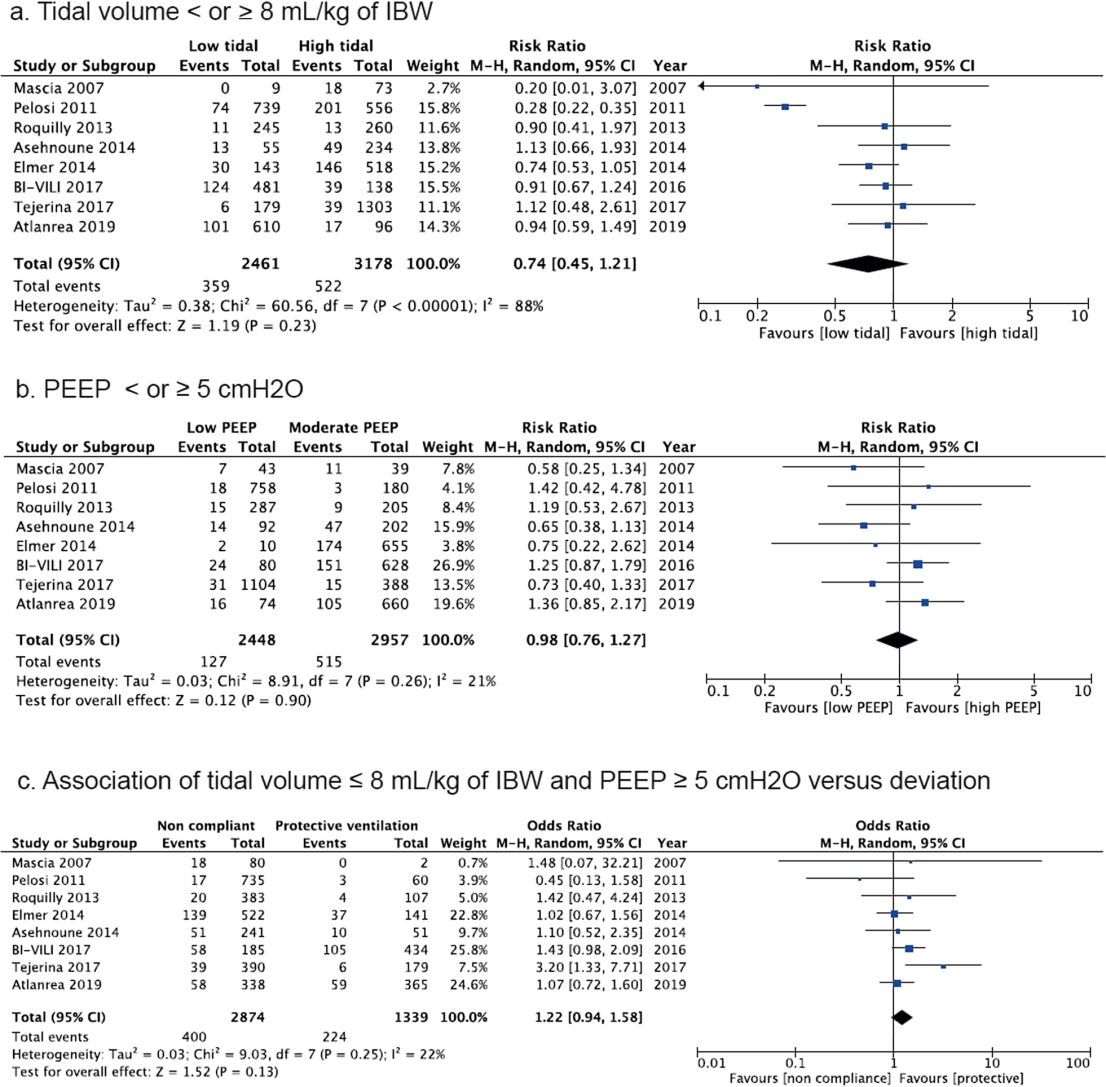
Development of ARDS according to ventilator settings

Low Vt compared to high Vt (359/2461 [14.6%] vs. 522/3178 [16.4%]; OR 0.74 (95% CI 0.45 to 1.21), *p* = 0.23, *I*^2^ = 88% Fig. 2a) as well as low PEEP compared to moderate to high PEEP (515/2957 [17.4%] vs. 127/2448 [28.3%], OR 0.98 (95% CI 0.76 to 1.26), *p* = 0.9, *I*^2^ = 21% Fig. 2b) did not decrease the risk of ARDS. There was no association between the risk of ARDS and protective ventilation strategy (224/1339 [16.7%] vs. 400/2874 [13.9%], OR 1.22 (95% CI 0.94 to 1.58), *p* = 0.13, *I*^2^ = 22% Fig. 2c).

**Fig. 3:**
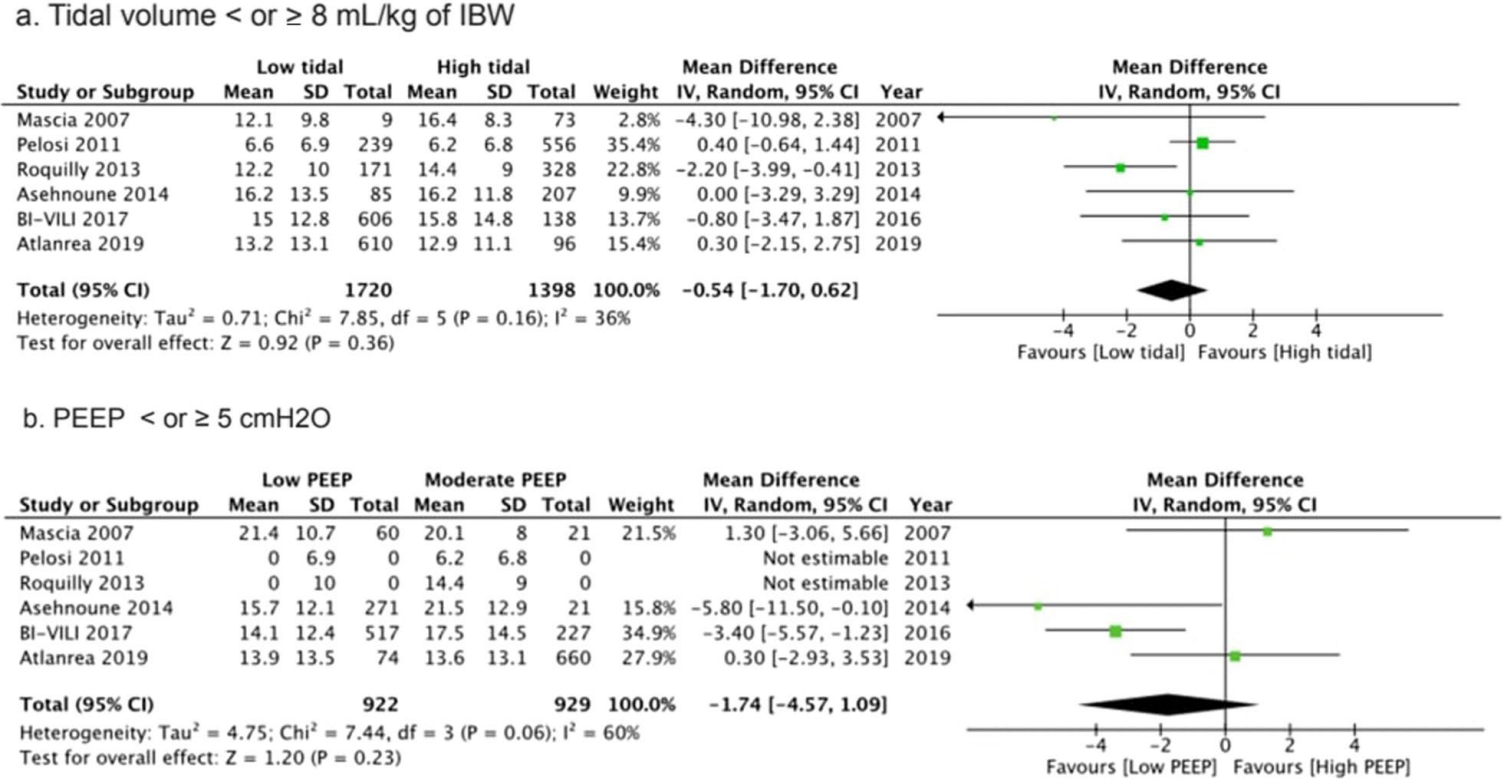
Duration of mechanical ventilation according to ventilation setting

The duration of mechanical ventilation was not reduced in the intervention group, low tidal versus high tidal volume (WMD − 0.54 days (95% CI − 1.7 to + 0.62); *p* = 0.36, *I*^2^ = 36%, (Fig. 3a). Low PEEP compared to High PEEP did not reduce the duration of mechanical ventilation (WMD-1.74 days (95% CI − 4.57 to + 1.09); *p* = 0.06, *I*^2^ = 60%, (Fig. 3b).

**Fig. 4:**
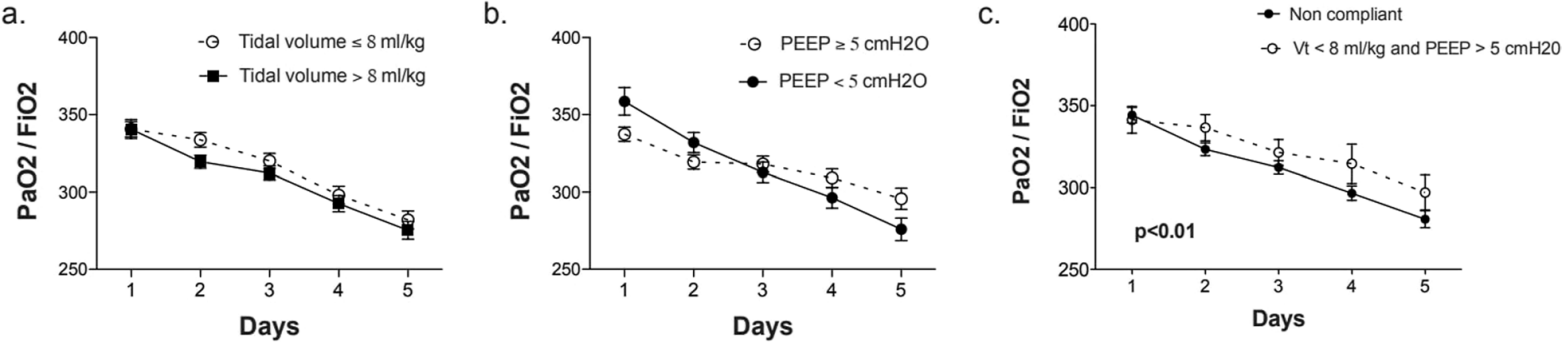
Effect of low tidal volume and moderate positive end-expiratory pressure on arterial oxygenation during the 5 first days

The time course of PaO2/FiO2 ratio over the first 5 days was not different between patients revising low Vt vs. high Vt; or low PEEP vs. moderate to high PEEP (Fig. 4a, b). However, a protective ventilation strategy improved PaO_2_/FiO_2_ ratio in the first 5 days of mechanical ventilation (*p* < 0.01 for group and time effects, not significant for time-treatment interaction, Fig. 4c).

## Discussion

In this systematic review exploring the role of mechanical ventilation in patients with acute BI, we found that: (1) low Vt, moderate to high PEEP levels and protective ventilation strategy (low Vt and moderate to high PEEP levels) are not associated with reduced in-hospital mortality or lower risk of ARDS; (2) a protective ventilation strategy was associated with a higher PaO2/FIO2 ratio over the first 5 days of hospitalisation.

The strength of our analysis relies on the fact that no clear evidence on optimal ventilation settings in patients with acute BI is currently available. The recently published international recommendations on the settings of mechanical ventilation strategies in this population remain undetailed regarding using these strategies, especially when intracranial pressure is unstable [16]. Further, we included many patients with acute BI from studies with detailed mechanical ventilation settings.

Acute BI are a growing healthcare issue. In our study, most patients had TBI and haemorrhagic stroke (mainly subarachnoid haemorrhage) with an overall in-hospital or 28-day mortality of 44.3%. In severe TBI patients, the reported mortality rate is high, ranging from 30 to 40%, and survivors experience a high burden of physical and cognitive disabilities profoundly impacting the lives of patients and family members with increased costs for society [17, 18].

MV may induce an inflammatory response in the lungs promoting remote organ failure [19]. In patients with ARDS, low Vt associated with moderate to high levels of PEEP has been shown to improve outcomes [20, 21]. During the last decade, substantial evidence has emerged showing that the brain modifies pulmonary responses to physical and biological stimuli by various mechanisms, including the modulation of neuroinflammatory reflexes and the onset of abnormal breathing patterns [22]. This hazardous crosstalk between the lungs and brain indicates that ameliorating lung functions may impact the neurologic outcome, and an accurate ventilation strategy may probably decrease long-term disabilities [23]. Further, patients with ABI are at increased risk of pulmonary complications [24]. There is no consensus on how to ventilate patients with acute brain injury. A practice survey of the European Society of Intensive Care Medicine has shown that in patients with ABI, the ventilatory management, targets and practice of adult severe TBI patients with and without respiratory failure are widely different among centres [11] and significantly depend on local policies and clinical practice.

The last update of the international recommendations on TBI patients does not specifically address mechanical ventilation, and strict control of PaCO2 to avoid hypercapnia is the only factor mentioned. Indeed, in the early phase after severe brain injury, if the PaCO2 is not tightly controlled, the intracranial pressure may rise to unacceptable levels. Historically, to maintain PaCO2 at an acceptable level, the tidal volume was set to high values (at or above 10 ml/kg), and the PEEP level was set to low levels or ZEEP. However, respiratory complications, including bacterial pneumonia, pulmonary oedema or ARDS, remain a significant cause of poor outcomes in brain-injured patients. In an observational study on 576 patients, Wartenberg et al. have shown that pulmonary complications are independent risk factors for poor outcome [25]. Kahn et al., in another observational study, also suggested that acute lung injury is an independent risk factor for death in 620 SAH patients [26]. These data suggest that we should more strictly control the lung to protect the brain. Recent European Society of Intensive Care Medicine (ESICM) recommendations on mechanical ventilation in patients with acute BI suggested that ZEEP should be avoided in this population and PEEP should be set according to the same principles considered in the general ICU population. Experts generally suggest tidal volume and lung protective strategies, but no recommendations are provided in case of unstable intracranial hypertension [16].

We found that low VT, high to moderate PEEP, or a protective ventilation strategy combining both did not improve survival. This is in line with two recent randomized controlled trials in patients without ARDS but including a minority of neurological patients showing no beneficial effects of individual ventilatory settings with low Vt or moderate to high PEEP levels on clinically relevant outcomes [27, 28]. In patients undergoing surgery Vt up to 10 ml/kg IBW appears to be protective against postoperative pulmonary complications [29, 30]; but moderate to high PEEP [31] has not been found to be associated with less risk of postoperative pulmonary complications. Mascia et al. [32] have shown that the proportion of ALI/ARDS in 86 patients with ABI was directly proportional to the tidal volume applied. The percentage of ALI/ARDS was below 10% when the tidal volume was < 9 ml/kg per IBW and was above 30% when the tidal volume was above 11 ml/kg per IBW. In a retrospective study, Elmer et al. [33] confirmed these data by showing that high tidal volumes (> 10 ml/kg per IBW) were associated with death and ARDS in patients with stroke.

PEEP may increase ICP by increasing the intrathoracic pressure that may impair the venous return from the brain. The fear for increased ICP explains that in up to 80% of patients with BI a PEEP ≤ 5 cm H_2_O is delivered. However, in a retrospective study, the effects of PEEP on intracranial pressure were evaluated in 20 patients with ABI complicated by ALI/ARDS. From 0 to 15 cm H2O, the PEEP level alters neither intracranial pressure nor cerebral perfusion pressure. Also, the effect of PEEP on ICP is probably small if the PaCO2 is controlled [34]. If moderate PEEP level causes no harm, it may improve oxygenation since our group found that moderate PEEP i.e. levels (6–8 cm H_2_O) favourably altered the time-evolution of the PaO2/FIO_2_. It should also be mentioned that most patients with severe BI are monitored for intracranial pressure rendering PEEP titration safe. Protective mechanical ventilation combining low Vt and moderate to high levels of PEEP was effective in improving outcomes during surgery [35]. The efficacy of protective ventilation in severely brain-injured patients has been evaluated in two before-after studies involving 1243 patients [3, 10]. In the first trial performed in 2 ICUs, a tidal volume between 6 and 8 ml/kg of ideal body weight and PEEP > 3 cm H_2_O was applied. An increased number of ventilatory-free days was observed during the intervention period [3]. In the second multicenter nationwide study in 749 patients, a protective ventilation strategy (≤ 7 ml/kg of ideal body weight and a PEEP level between 6 and 8 cmH2O) associated with early extubation was evaluated [10]. In the sub-group of patients for which the two recommendations were applied, the number of ventilatory-free days at day 90 was enhanced, and the mortality rate was reduced. In both studies, compliance to a protective ventilation strategy did not impair outcome, or ICP provided PaCO_2_ was monitored and maintained to recommended values. These results offer simple and applicable data to the neuro-ICU physician on how to reach the goals of PaCO_2_ with a strategy of modifying respiratory rate rather than tidal volume. Indeed, a low tidal volume (7.0 ml/kg of IBW) provided little change in the level of PaCO2 [10]; these results are in line with those of a meta-analysis showing that low tidal volumes increase PaCO2 moderately (from 38 to 41 mmHg).

Our study has several limitations that hamper definitive conclusions. Firstly, the results must be interpreted within the context of non-randomized studies trials in the current meta-analysis. However, the studied populations are reasonably homogeneous. Indeed, even if the cerebral lesion's mechanism differs, the mechanical ventilation's impact remains the same for all brain injured patients. We can note the low adherence to the set of measures in the 2 interventional studies [3, 10]. However, in the second study [10], we compared patients with incomplete adherence to all measures to those with complete adherence and mortality was lower in the latter group. Secondly, the studies were performed over an extensive range of years. In the first study [32] published in 2003, the average tidal volume was set at 9.6 ml/Kg of IBW and PEEP at 4.2 mmHg whereas in the interventional phase of the Asehnoune et al. study [10] published in 2014, the tidal volume was set at 7 ml/kg of IBW and 6.1 of PEEP; a secular trend is therefore probably not negligible and was not evaluated. The evolution of practices explains that the rate of ARDS, and the duration of mechanical ventilation have been reduced during the last 10 years and this may affect the extrapolation of our results. Thirdly, another limitation relies on the fact that the duration of hypoxemia, which could not be collected, is particularly relevant because it is associated with a poor outcome [37, 38]. However, in this same study [38], Robba et al. found a direct association between mortality and the PaO2/FiO2 ratio. Following these data, our results show an improved PaO2/FiO2 ratio in patients receiving protective ventilation. Fourthly, we did not assess the effect of mechanical ventilation settings on ICP or PaCO2. The impact of PCO2 is paramount in the management of head trauma. PaCO2 is one of the main parameters of systemic secondary brain insult, in the Bi-Vili study [10] we showed that ventilation above or below 7 ml/kg did not influence the PaCO2 within the first 5 days, as did PEEP levels above or below 5 cmH2O. It would have been interesting to identify patients for whom PaCO2 could not be set within the recommended ranges because of the occurrence of ARDS and significant alterations in lung compliance.

In conclusion, a protective ventilation strategy with low Vt and moderate to high PEEP does not improve outcome but improves oxygenation in mechanically ventilated patients with ABI. Further research is needed to assess the role of protective ventilation strategies in this population.

## Supplementary Information

**Additional file 1: Table S1** Prisma checklist. **Fig. S1**: Flow chart of the literature research for the meta-analysis. **Table S2** Quality Assessment of Eligible Studies. **Table S3** Detailed Characteristics of Eligible Published Studies.

## Data Availability

All data generated or analysed during this study are included in the published articles.

## Abbreviations

ARDS: Acute respiratory distress syndrome
ICU: Intensive care unit
ABI: Acute brain injury
PBW: Predicted body weight
IBW: Ideal body weight
ALI: Acute lung injury
ICP: Intra-cranial pressure
TBI: Traumatic brain injury
SAH: Subarachnoid haemorrhage
PEEP/ZEEP =: Positive/zero end expiratory pressure
